# A Case of Mucopolysaccharidosis II Caused by a Novel Variant with Skin Linear Hyperpigmented Streaks along Blaschko’s Lines

**DOI:** 10.3390/ijms24065647

**Published:** 2023-03-15

**Authors:** Viktoriia Sofronova, Elizaveta Gurinova, Diana Petukhova, Hiroko Fukamatsu, Takenobu Yamamoto, Yumi Aoyama, Polina Golikova, Gavril Moskvitin, Roza Ivanova, Mira Savvina, Filipp Vasilev, Takahito Moriwaki, Seigo Terawaki, Aitalina Sukhomyasova, Nadezhda Maksimova, Takanobu Otomo

**Affiliations:** 1 Department of Molecular and Genetic Medicine, Kawasaki Medical School, Kurashiki 701-0192, Japan; 2 Laboratory of Molecular Medicine and Human Genetics, North-Eastern Federal University, 677013 Yakutsk, Russia; 3 Medical Genetics Center, Republic Hospital No. 1—National Center of Medicine, 677019 Yakutsk, Russia; 4 Department of Dermatology, Kawasaki Medical School, Kurashiki 701-0192, Japan

**Keywords:** mucopolysaccharidosis II, Hunter syndrome, skin pigmentation, Blaschko’s line

## Abstract

We report a case of an eight-year-old boy with mucopolysaccharidosis (MPS) II with atypical skin lesions of hyperpigmented streaks along Blaschko’s lines. This case presented with mild symptoms of MPS such as hepatosplenomegaly, joint stiffness, and quite mild bone deformity, which was the reason for the delay in diagnosis until the age of seven years. However, he showed an intellectual disability that did not meet the diagnostic criteria for an attenuated form of MPS II. Iduronate 2-sulfatase activity was reduced. Clinical exome sequencing of DNA from peripheral blood revealed a novel pathogenic missense variant (NM_000202.8(IDS_v001):c.703C>A, p.(Pro235Thr)) in the *IDS* gene, which was confirmed in the mother with a heterozygous state. His brownish skin lesions differed from the Mongolian blue spots or “pebbling” of the skin that are observed in MPS II.

## 1. Introduction

Mucopolysaccharidosis type II (MPS II, Hunter syndrome, OMIM #309900) is an X-linked recessive lysosomal storage disorder caused by variants in the *IDS* gene. The pathogenic *IDS* variant leads to a deficiency of the lysosomal enzyme iduronate 2-sulfatase and accumulation of glycosaminoglycans (GAG) such as dermatan sulfate (DS) and heparan sulfate (HS) that causes impaired cellular functions and multiple organ damage [1,2]. MPS II is characterized by a wide spectrum of clinical manifestations including intellectual disability, distinctive facial features, skeletal deformities called “dysostosis multiplex,” short stature, joint stiffness, hepatosplenomegaly, inguinal and umbilical hernias, extensive Mongolian blue spots (nevus), cardiorespiratory abnormalities such as thickening of the heart valves and upper airway tissues [3]. Based on the onset and severity of the symptoms, MPS II is classified into two subtypes, namely, the early progressive (severe) form, which shows severe symptoms including neurologic decline and death usually in the second decade of life, and the slowly progressive (attenuated) form, which does not have intellectual impairment [1,3]. MPS II is diagnosed by an abnormal qualitative and quantitative pattern of GAG in urine, reduced iduronate 2-sulfatase activity in lymphocytes or fibroblasts, and genetic testing [4]. More than 600 pathogenic variants in the *IDS* gene have been described worldwide [5]. Skin abnormalities may also indicate the presence of MPS II. Extensive Mongolian blue spots are observed in patients of Asian, Native American, and African descent from birth when other symptoms are not yet apparent [6]. Some patients develop “pebbling” of the skin, which is described as firm skin with ivory white papules and nodules, and is pathognomonic of MPS II [7,8].

In this report, we describe a male patient with MPS II caused by a novel missense variant in the *IDS* gene. He showed reduced iduronate 2-sulfatase activity and intellectual disability; however, other systemic clinical manifestations such as hepatosplenomegaly, joint stiffness, and bone deformity were mild for MPS II, which delayed the clinical diagnosis of the disease until seven years old. He exhibited hyperpigmented streaks along Blaschko’s lines in his skin, which was atypical for MPS II.

## 2. Results

### Case Presentation

**Figure 1:**
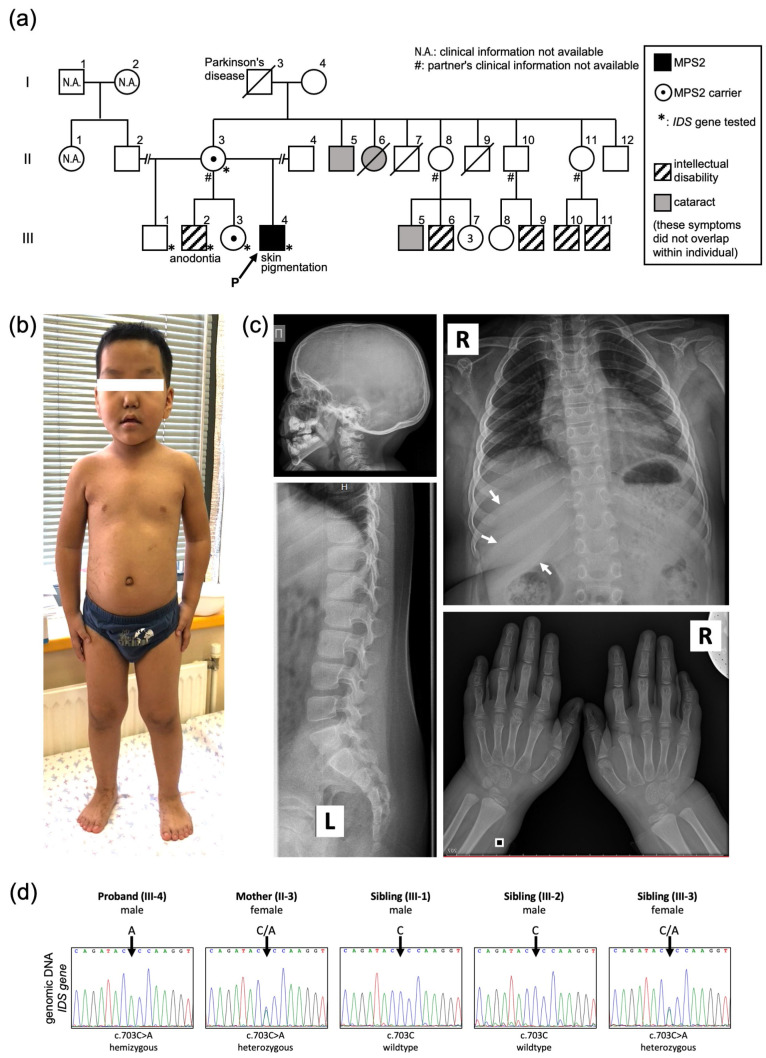
Pedigree, clinical pictures, and genetic diagnosis of MPS II case. (**a**) Family tree of the case. Genetic testing of *IDS* was performed on II-3, III-1, 2, 3, and 4 (indicated with *). Causes of death were complications from Parkinson’s disease (I-3 at 77 years old), infection (II-6 at 2 months old), and electrical accident (II-7 at 6 years old). (**b**) Whole body image of the proband aged five years and seven months. Slight distinctive facial features, including prominent forehead, broad nasal bridge and hypertelorism, were seen. (**c**) Radiographic images of the proband at the age of seven years. The characteristic finding was absent except for slight widening “oar-shaped“ ribs (indicated by arrows). (**d**) Genetic test of *IDS* gene with Sanger sequencing. A missense variant c.703C>A was identified in the proband (III-4), proband’s female sibling (III-3), and mother (II-3).

The pedigree is shown in Figure 1a. The proband (III-4) is an eight-year-old Yakut boy, who is the fourth child of healthy unrelated Yakut parents. His mother (II-3) has four children (III-1 to 4) with three different partners. A slight decrease in intelligence and partial anodontia in the upper jaw was noticed in the proband’s half-sibling (III-2), but the diagnosis was not confirmed. Some cases of intellectual disability or cataracts were reported by the mother’s relatives, however, detailed medical information and confirmative testing are unavailable to them.

There was no particular problem with the maternal pregnancy except for an episode of slight bleeding in the first trimester of pregnancy, which might be an indication of impending miscarriage and recovery. He was born at 36 weeks of gestation by natural childbirth, with a weight of 3138 g (97 percentiles), height of 51 cm (97 percentiles), and Apgar scores of 6/8. At his birth, his mother was 28 years old and his father was 27 years old.

At the age of one month, the proband was consulted by a geneticist when pronounced parietal tubercles and hypertrichosis of the body were noticed. Symmetrically located spots of light brown color, like “confetti” splashes, were found in the armpit, the neck, the stomach, and on both feet, extending to the thighs in a linear streak pattern. According to the mother’s statement, these skin lesions began about two weeks after birth. At the age of one year and three months, gross delay in psycho-speech development and hypertrophy of the adenoids, which caused difficulties in nasal breathing, and snoring were diagnosed. Epilepsy appeared at the age of three: episodes would start with him gazing at a single point, and lead to a myotonic seizure of the extremities lasting about 10–15 min, after which he would fall asleep. The epilepsy was not treated with anticonvulsants due to the refusal of medication by the family. Seizures occurred every three months but stopped appearing at age seven. Around this time, mild joint stiffness of the fingers was observed. Developmental milestones were delayed: head control at 13 months, sitting without support at 15 months, and walking without support at 30 months. Intellectual ability was assessed at the age of seven years by the Wechsler Intelligence Scale for Children (WISC, Russian adapted version [9]), Verbal IQ = 47, performance IQ = 43, and full-scale IQ = 39, which corresponds to a severe intellectual disability with this version. His karyotype was normal (46, XY, in 15/15 cells) by G-banding.

**Figure 2:**
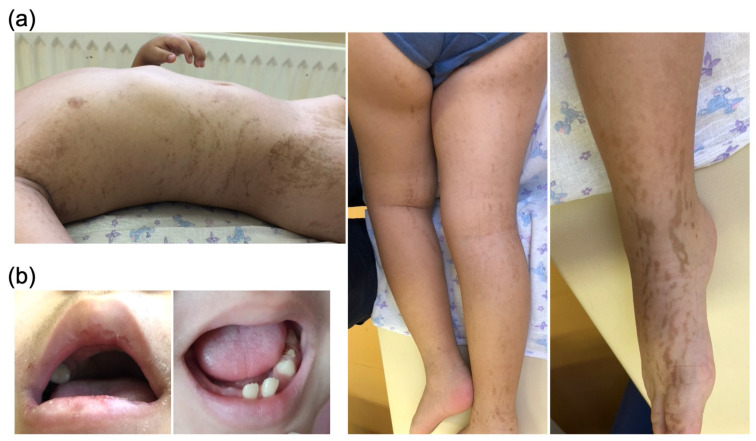
Atypical findings of MPS II found in the proband. (**a**) Skin linear hyperpigmented streaks along Blaschko’s lines. (**b**) Anodontia and small teeth.

Mucopolysaccharidosis (MPS) was suspected at the age of seven years after the recognition of the combined distinct features such as intellectual disability, hepatosplenomegaly, joint stiffness, and hypertrophy of adenoids together with the distinctive facial features, including a prominent forehead, flat nasal bridge, and hypertelorism (Figure 1b). Anodontia and small teeth were observed (Figure 2b). His skin was swarthy and exhibited hypertrichosis on his back and lumbar region. Linear hyperpigmented streaks (which were already present at the age of one month) persisted on his limbs, armpit, and lateral abdomen along Blaschko’s lines (Figure 2a). The radiographic evaluation of bone revealed slight thickening of the ribs; however, other changes of dysostosis multiplex were absent (Figure 1c). Biochemical blood tests were normal. Ophthalmologic investigation revealed myopia of mild degree and compound myopic astigmatism of both eyes and strabismus, although corneal clouding was not observed. At the age of eight years, his height was 127.5 cm (75 percentiles), and his weight was 41.9 kg (97 percentiles). 

Due to geographical factors of the Sakha Republic (Yakutia) regarding specimen delivery, it was not easy to measure urinary GAG using fresh urine. Lysosomal enzyme activity was measured with dried blood spot and showed low activity of iduronate 2-sulfatases at 2.48 µmol/L/hour (reference range from 10 to 50), and he was diagnosed as MPS II. Clinical exome sequencing on DNA from the proband’s peripheral blood was performed, which found that the proband was hemizygous for the novel missense variant (NM_000202.8(IDS_v001):c.703C>A, p.(Pro235Thr)) in exon 5 of the *IDS* gene. This variant was not registered in the databases of gnomAD, 1000 Genomes Project, and ClinVar. In silico prediction tools were used for estimating pathogenicity: MutationTaster (Tree vote: 95|5, “deleterious”); MutPred2 (score = 0.875, “pathogenic”); PolyPhen2-HDIV (score = 1.0, “probably damaging”); and PolyPhen2-HVAR (score = 1.0, “probably damaging”). Altogether, according to the ACMG guideline in 2015 [10], this sequence variant was interpreted as “likely pathogenic” (PS3, PM2, PP3).

Clinical exome sequencing was performed only with the proband. The newly found missense variant was confirmed by direct Sanger sequencing in the proband (III-4), and also confirmed that his mother (II-3) and one half-sibling (III-3) were heterozygous carriers of this variant (Figure 1d).

In the public health check-up system for children (performed monthly in the first year of life, then at 15 months, 18 months, and annually after 2 years of age), no abnormal heart findings requiring detailed examination or treatment were noted. Echocardiography was performed after the diagnosis of MPS II (at 8 years old). Dilatation was not observed in the left and right ventricle or atrium, aorta, ascending aorta, or pulmonary artery. The diastolic left ventricular posterior wall thickness was 6 mm (median; 7 mm, 2nd/98th percentiles; 5/9 mm) [11], and the ejection fraction was 72% (median; 71%, 2nd/98th percentiles; 62/79%) [11], both were considered within the normal range. No thickening of the mitral, tricuspid or pulmonary valves was observed, however, mild mitral regurgitation (Grade I) [12] was noted. At this point, no specific treatment was deemed necessary, and the patient was placed under careful observation. After the diagnosis, the proband started treatment with intravenous enzyme replacement therapy by Hunterase (Idursulfase beta). After one year of enzyme replacement therapy, no change has been observed so far in his clinical course.

## 3. Discussion

MPS are characterized by distinctive facial features, hepatosplenomegaly, multiple bone deformities called dysostosis multiplex, excessive urinary GAG, and so on. Accumulation of GAG leads to tissue damage in different enzymes and pathogenic variants in the causative genes. In this study, MPS was suspected in this seven-year-old due to hepatosplenomegaly, mild joint stiffness, and intellectual disability. The low enzyme activity of lysosomal iduronate 2-sulfatase confirmed MPS type II, as well as a pathogenic variant in the *IDS* gene. Radiographic bone changes of dysostosis multiplex were not obvious with the exception of the thickening of ribs (Figure 1c). On the other hand, the neurological involvement included a delay in developmental milestones and seizures, which seems more severe compared with the physical manifestations.

A characteristic feature of this case was a skin lesion. Some patients with MPS II develop ivory-colored skin lesions or extensive Mongolian blue spots as pathognomonic of MPS II [6,7,8]. However, this case showed brownish skin linear hyperpigmented streaks along Blaschko’s lines (Figure 2a), atypical of MPS II. Blaschko’s lines map out a migratory history of ectodermal skin cells after their proliferation and movement from embryonic development into the fully formed organism [13]; further, skin disorders following these lines may be explained by the mosaicism due to X-chromosome inactivation or early somatic mutations [14].

This characteristic skin lesion suggests a skin disease called incontinentia pigmenti (IP, Bloch-Sulzberger syndrome, OMIM #308300). IP is caused by pathogenic variants in the *IKBKG* (*NEMO*) gene on the X-chromosome, and characterized by skin lesions (hyperpigmented streaks), ophthalmological (optic atrophy, retinal detachment), odontologic (partial anodontia) and neurological (epilepsy, psychomotor delay) symptoms [15,16]. In addition to skin pigmentation, this case exhibited atypical symptoms for MPS II in the teeth (hypodontia, microdontia, abnormal shape) and eyes (strabismus) that are observed in IP (Table 1). Skin lesions change over the years from stage I to IV in the clinical course of IP [15]; however, his hyperpigmentation started just after his birth as “confetti” splashes and grew into streaks (like stage III lesions in IP)—and continued over eight years, which is not a typical clinical course for IP. Since IP is a disorder of X-linked dominance, a male with a pathogenic variant in the *IKBKG* gene is embryonically lethal, except for a few cases of male patients with mosaicism of the wild type and a pathogenic *IKBKG* gene or with Klinefelter syndrome (47, XXY) [17,18,19,20,21]. In this case, the proband’s karyotype was normal by G-banding, and mosaicism was not detected with clinical exome sequencing using peripheral blood. Additionally, we obtained genomic DNA from the skin with/without hyperpigmented lesion, however, the PCR method could not confirm the presence of the deletion of exons 4–10 in the *IKBKG* gene (Figure S1) that is reported at a mosaic state in male IP patients [17,20].

The other suspected disorder from the proband’s clinical manifestations is X-linked syndromic intellectual developmental disorder with pigmentary mosaicism and coarse facies (MRXSPF, OMIM #301066). Recently, de novo variants in the transcription enhancer factor 3 (*TFE3*) gene associated with intellectual disability, skin pigmentation, and lysosomal storage-like features were reported (Table 1) [22,23]. They reported 17 cases, including 12 female and 5 male patients. Female cases have pathogenic missense or splicing variants in their one allele at heterozygous states. All five male cases have missense variants, however, molecular investigations revealed mosaicism in two of five male patients: 65% or 88% of reads contained pathogenic variants. TFE3 is a transcription factor that belongs to the melanocyte inducing transcription factor (MITF) family together with transcription factor EB (TFEB) and transcription factor EC (TFEC). These molecules function in various biological pathways, including lysosomal signaling and the mechanistic target of rapamycin (mTOR) [24]. Clinical features of MRXSPF include skin pigmentation on Blaschko’s lines, severe intellectual disability, and coarse facial features, which overlaps with this case (Table 1). The full coding sequence of the *TFE3* gene was analyzed for this case, but no mutation or mosaic condition was found, even with sufficient reading depth.

The absence of preceding inflammation and vesicular lesions on the linear hyperpigmented area raises the possibility of a rare sporadic pigmentary skin disease, linear and whorled nevoid hypermelanosis (LWNH). Extracutaneous findings including neurological, skeletal, and ocular anomalies have been reported in LWNH [25,26,27], which partially overlaps with our case with intellectual disability. The pathomechanism of LWNH is hypothesized to be related to an abnormality during embryogenesis with somatic mosaicism [28]. The causative gene for LWNH is not identified, although a familial case and the involvement of X-chromosomal mosaicism have been reported [14].

Relatively severe intellectual disability compared with the physical severity of MPS II observed in this case may suggest an association of the *IKBKG* or *TFE3* genes or a complication of LWNH. Although clinical manifestations of these diseases partially overlap with the present case, molecular diagnostic results did not allow us to conclude the involvement of these genes in addition to the pathogenic *IDS* variant, because the possible effect of these genes, with an undetectable level of mosaicism, cannot be excluded, which we realize is a limitation of this study. We have analyzed the proband’s clinical exome sequencing data, and only the *IDS* variant was confirmed as pathogenic for his clinical manifestations. Indeed, we focused on several genes other than *IKBKG* and *TFE3,* which may be potentially involved in skin lesions, but no variant was confirmed.

Finally, some family members related to the proband’s mother (II-3) complain of intellectual disability or cataracts (Figure 1a). There is a possibility that these symptoms are caused by some genetic reasons of the family, and this genetic background may have affected the symptoms of the proband through his mother. Because clinical exome sequencing was performed only with the proband, not with the parents or siblings in this case, the involvement of modulative gene variants other than *IDS* cannot be confirmed.

In conclusion, we have identified a novel variant c.703C>A, p.(Pro235Thr) in the IDS gene as a cause of MPS II. Systemic manifestations such as the hepatosplenomegaly, joint stiffness and bone deformities, were mild, but consistent with those of MPS II together with the pathogenic variant of the *IDS* gene and reduced iduronate 2-sulfatase activity. On the other hand, pigmentation along Blaschko’s line was not a typical manifestation of MPS II. Since skin lesions along Blaschko’s line are commonly attributed to random inactivation of the X-chromosomal gene in females, or mosaicism of the genetic variant, we speculate that the skin lesion is caused independently of MPS II. We tested the proband for genes that have been reported to be associates with such hyperpigmentation, however, were unable to conclude them as the cause. The probands’ relatives also presented with a variety of symptoms, and it may be necessary to perform CES on a large scale and on a wide range of relatives in order to centrally explain their causes.

## 4. Materials and Methods

### Molecular Diagnosis

Clinical exome sequencing was carried out using a MiSeq sequencer (Illumina, San Diego, CA, USA) on the proband. Genomic DNA was collected from peripheral blood for genetic testing. For sample preparation, the technique of selective capture of DNA sites belonging to the coding regions of 4490 genes with known clinical significance was used (Clinical Exome Solution by Sophia Genetics V2 for Illumina MiSeq, SOPHiA GENETICS, BS.2195.0102-16, Switzerland). The quality of the readings was assessed using the FastQC program, and the removal of adapters and trimming of the sequence was performed with the Trimmomatic-0.36 software package. Alignment of the readings to the reference sequence of the human genome (GRCh37/hg19) was carried out using Bowtie2. Population databases gnomAD (https://gnomad.broadinstitute.org (accessed on 13 March 2023)), 1000 Genomes Project (https://www.internationalgenome.org (accessed on 13 March 2023)), and disease database ClinVar (https://www.ncbi.nlm.nih.gov/clinvar/ (accessed on 13 March 2023)) were used to obtain the frequencies of the variant in a large population. In silico algorithms, MutationTaster2021 (https://www.genecascade.org/MutationTaster2021/ (accessed on 13 March 2023)), MutPred2 (http://mutpred.mutdb.org/ (accessed on 13 March 2023)), and PolyPhen-2 (http://genetics.bwh.harvard.edu/pph2/ (accessed on 13 March 2023)) were used to predict the pathogenicity of the variant. Sanger sequencing was performed following genomic PCR using specific primers: fw 5′—GACATGTCGAGGGCCAGATG—3′ and rv 5′—CTTACATGTTCTTGGCCTTGAC—3′ with DNA from peripheral blood samples of the proband, mother and siblings.

## Figures and Tables

**Table 1:** Comparative table of the characteristics of three disorders: Mucopolysaccharidosis type II (MPS II), Incontinentia pigmenti (IP), X-linked syndromic intellectual developmental disorder with pigmentary mosaicism and coarse facies (MRXSPF).

Disease (OMIM)	MPS II (#309900) [1,3]	IP (#308300) [15]	MRXSPF (#301066) [22,23]
Gene	*IDS*	*IKBKG/NEMO*	*TFE3*
Locus	Xq28	Xq28	Xp11.23
Inheritance	X-linked recessive	X-linked dominant	X-linked
Dermatological	Ivory-colored skin lesions Extensive blue nevus (Mongolian spots)	Skin lesions (stages I-IV): I. Erythema II. Verrucous lesions III.Hyperpigmented streaks and whorls (Blaschko’s line) IV. Pale, hairless, atrophic linear streaks	Blaschkoid pigmentary
Nervous	Intellectual disability Developmental delay Behavior difficulties	Intellectual disability CNS vasculopathy Seizures	Intellectual disability Developmental delay Epilepsy
Skeletal	Dysostosis multiplex (J-shaped sella turcica, bullet-shaped phalanges, metacarpal pointing, metaphyseal widening of the long bones with cortical thinning, oar-shaped ribs, flared iliac wings, round- or hooked-shape vertebral bodies) Joint contracture Hip dysplasia	N.D.	Thickening of ribs Hyperlordosis Scoliosis Hip dislocation Limitation of elbow extension
Facial dysmorphism	Prominent forehead Flat nasal bridge Flared nostrils Synophrys Hypertelorism Thick lips Macroglossia	N.D.	Flat nasal bridge Short nose with anteverted nares Widely spaced and almond-shaped eyes Thick lips Facial hypertrichosis
Odontologic	Irregular shaped teeth Overgrown gingival tissue	Anodontia Microdontia Abnormally shaped teeth Delayed eruption or impaction	N.D.
Ophthalmological	Optic nerve atrophy Retinopathy	Strabismus Microphthalmia Retinal detachments Cataracts Optic atrophy Retinal pigmentation	Strabismus Retinal degeneration Depigmented macula of the iris Oculomotor apraxia
Cardiovascular	Valvular disease Cardiomyopathy Rhythm disorder	N.D.	Congenital heart defect
Gastrointestinal	Hepatosplenomegaly Umbilical/inguinal hernia Chronic diarrhea	N.D.	Umbilical hernia Anteriorly displaced anus
Laboratory test	Hypersecretion of glycosaminoglycan in urine * Low/absent enzyme activity of iduronate 2-sulfatase	N.D.	N.D.

Underlined symptoms were observed in the proband of this study. N.D., not described. *: urinary glycosaminoglycan was not measured in this case.

## Data Availability

All relevant data, which support the findings of the study, are within the manuscript.

## Supplementary Materials

The following supporting information can be downloaded at: https://www.mdpi.com/article/10.3390/ijms24065647/s1.
