# Supplementary material for: A Case of Mucopolysaccharidosis II Caused by a Novel Variant with Skin Linear Hyperpigmented Streaks along Blaschko’s Lines
Source: Int J Mol Sci. 2023 Mar 15;24(6):5647. doi: 10.3390/ijms24065647 (PMC10057584; doi:10.3390/ijms24065647)
Supplement: Supplementary file 1 [file ijms-24-05647-s001.zip › ijms-2251302-supplementary.pdf]

Supplementary Materials

A Case of Mucopolysaccharidosis II Caused by a Novel Variant with Skin Linear Hyperpigmented Streaks along Blaschko's Lines

Viktoriia Sofronova [1,2,†], Elizaveta Gurinova [3,†], Diana Petukhova [2], Hiroko Fukamatsu [4], Takenobu Yamamoto [4], Yumi Aoyama [4], Polina Golikova [2,3], Gavril Moskvitin [2,3], Roza Ivanova [2,3], Mira Savvina [2], Filipp Vasilev [2], Takahito Moriwaki [1], Seigo Terawaki [1], Aitalina Sukhomyasova [2,3], Nadezhda Maksimova [2], and Takanobu Otomo [1,*]

[1] Department of Molecular and Genetic Medicine, Kawasaki Medical School, Kurashiki 701-0192, Japan;
[2] Laboratory of Molecular Medicine and Human Genetics, North-Eastern Federal University, 677013 Yakutsk, Russia
[3] Medical Genetics Center, Republic Hospital No. 1 – National Center of Medicine, 677019 Yakutsk, Russia
[4] Department of Dermatology, Kawasaki Medical School, Kurashiki 701-0192, Japan
* Correspondence: otomo@med.kawasaki-m.ac.jp; Tel.: +81-86-462-1111
† These authors contributed equally to this work

Figure S1. PCR analysis to detect presence of wild-type and deletion of exons 4-10.

(a)

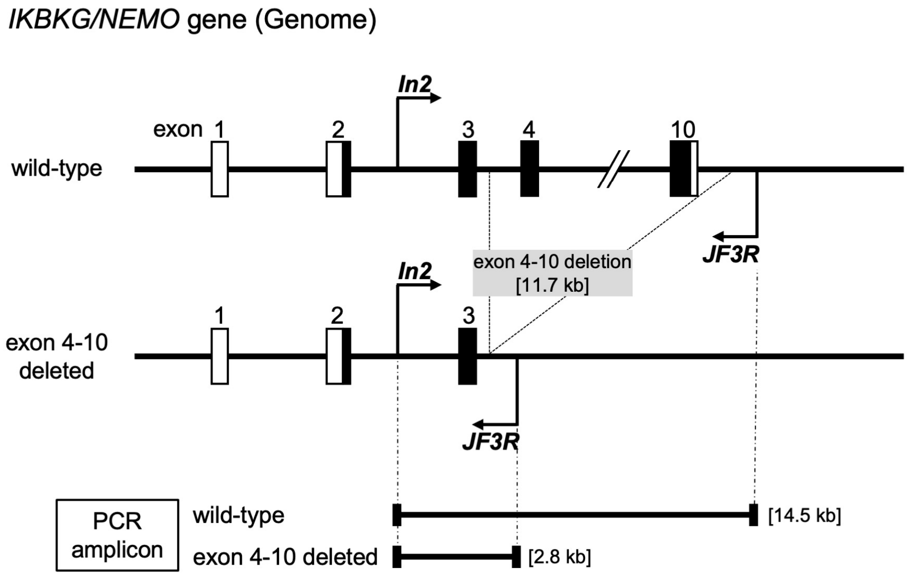

(b)

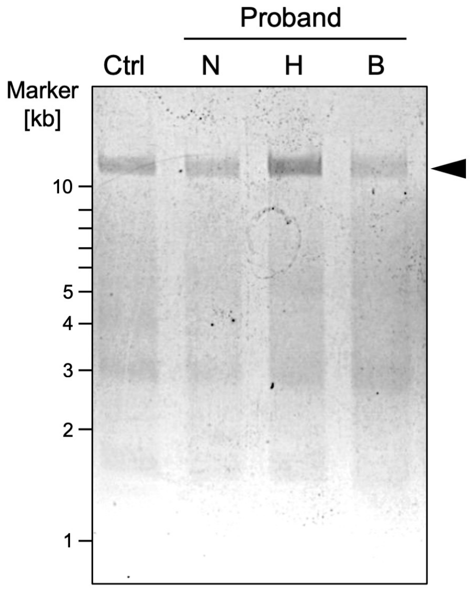

Figure S1 Schematic representation of *IKBKG/NEMO* gene and PCR results using primer set to detect exons 4-10 deletion.

(**a**) Structure of *IKBKG/NEMO* gene and the position of *IKBKG/NEMO* gene-specific primer set which was previously reported for the detection of exon 4-10 deletion [15]: forward primer *In2* and reverse primer *JF3R*. Expected PCR products with sizes 14.5 kb and 2.8 kb are derived from intact allele and exons 4-10 deleted allele, respectively. Coding exons are shown as black boxes. (**b**) Agarose gel electrophoresis of PCR products. PCR was performed using primers (*In2*/*JF3R*) with genomic DNA. Exon 4-10 deletion was not observed with this method. Ctrl: normal skin fibroblast, N: proband's skin fibroblast established from a non-pigmented skin part, H: proband's skin fibroblast established from a hyperpigmented skin lesion, B: proband's peripheral blood. Arrowhead indicates a 14.5 kb PCR amplicon corresponding to the intact allele. Marker; 1 kb ladder.
